# A cross-country qualitative study on contraceptive method mix: contraceptive decisionmaking among youth

**DOI:** 10.1186/s12978-021-01160-5

**Published:** 2021-05-25

**Authors:** Lynette Ouma, Burcu Bozkurt, Jill Chanley, Christine Power, Ronald Kakonge, Oluwatosin C. Adeyemi, Ramya Jawahar Kudekallu, Elizabeth Leahy Madsen

**Affiliations:** 1 International Youth Alliance for Family Planning, Washington, DC USA; 2 Department of Health Policy and Management, Gillings School of Global Public Health, University of North Carolina at Chapel Hill, Chapel Hill, NC USA; 3 PRB, Washington, DC USA; 4 University of Lagos, Lagos, Nigeria

**Keywords:** Method mix, Youth, Contraceptive decisionmaking, Family planning, Full range of methods

## Abstract

**Background:**

Youth ages 15 to 24, who comprise a large portion of sub-Saharan Africa, face a higher burden of unmet contraceptive need than adults. Despite increased international and national commitments to improving young people’s access to contraception, significant barriers impede their access to a full range of methods. To further explore these barriers among youth in Kenya, Nigeria, and Uganda, we conducted a qualitative study to capture the challenges that affect contraceptive method decisionmaking and complicate youth access to the full method mix.

**Methods:**

To understand factors that impact young people’s contraceptive decisionmaking process across all three countries, we conducted a total of 35 focus group discussions with 171 youth ages 15 to 24 and 130 in-depth interviews with key stakeholders working in youth family planning. Questionnaires aligned with the High Impact Practices in Family Planning’s elements of adolescent-friendly contraceptive services. Data were coded with MAXQDA and analyzed using a framework for contraceptive decisionmaking to identify relevant patterns and themes.

**Results:**

In all three countries, youth reported that condoms are the most commonly sought contraceptive method because they are easiest to access and because youth have limited knowledge of other methods. Youth from diverse settings shared uncertainty and concern about the safety and side effects of many methods other than condoms, complicating their ability to take full advantage of other available methods. While most youth in Kenya, Nigeria, and Uganda reported at least moderate confidence in obtaining the information needed to help choose a method, and only a few youth reported that they are completely unable to access contraceptives, other barriers still present a major deterrent for youth, including cost, inconvenient facility hours and long wait times, and stigma from family, community members, and providers.

**Conclusions:**

Young people’s ability to fully exercise their method choice remains limited despite availability of services, leading them to take the path of least resistance. Program implementers and policymakers should consider the diverse and often interconnected barriers that youth face in attempting to enjoy the benefits of a full spectrum of contraceptive methods and design multi-level interventions to mitigate such barriers.

**Supplementary Information:**

The online version contains supplementary material available at 10.1186/s12978-021-01160-5.

## Plain Language Summary

Despite increased international and national commitments to improving young people’s access to contraception, youth ages 15 to 24 face significant barriers to accessing a full range of contraceptive methods. This study conducted in-depth interviews with key stakeholders and focus group discussions with youth in Kenya, Nigeria, and Uganda to understand what factors impact youth’s decision to use or not use certain contraceptive methods. In all three countries, youth reported that condoms are the most commonly sought contraceptive method because they are easiest to access and because youth have limited knowledge of other methods. Youth from diverse settings shared uncertainty and concern about the safety and side effects of many methods other than condoms. While most youth in Kenya, Nigeria, and Uganda reported at least moderate confidence in obtaining the information needed to help choose a method, other barriers like cost, inconvenient facility hours and long wait times, and stigma from family, community members, and providers still present a major deterrent for youth who want information on contraceptive methods. Program implementers and policymakers should consider the diverse and often interconnected barriers that youth face in attempting to enjoy the benefits of a full spectrum of contraceptive methods and design multi-level interventions to mitigate such barriers.

**Supplementary Information:**

The online version contains supplementary material available at 10.1186/s12978-021-01160-5.

## Background

As of 2020, one in every five people in sub-Saharan Africa (SSA) is between the ages of 15 and 24, and this population is expected to remain large over the next two decades [1]. Meeting the sexual and reproductive health needs of young people^1^ is critical to improving their overall health outcomes and confers multiple benefits to this population as they mature [2–4]. Yet this group continues to have high rates of teenage and unintended pregnancies, as well as a high percentage of unmet need, defined as women who would like to space or delay pregnancy but are not currently using a contraceptive method [5]. Offering youth a full range of contraceptive options, including long-acting and reversible contraceptives (LARCs) through adolescent-friendly contraceptive services is one of several promising elements of High-Impact Practices (HIPs) in Family Planning [6]. LARCs, like IUDs and implants, have higher efficacy and continuation among adolescents who choose them compared to short-acting methods [7–9]. Previous studies demonstrate that youth, including adolescents ages 15 to 19, will use a variety of methods, including LARCs, when offered a full range of choices in an enabling environment [10–13].

Kenya, Nigeria, and Uganda mirror the demographics of most other SSA countries, with young people ages 15 to 24 accounting for roughly 20% of their total populations [1]. At least one out of ten sexually active women ages 15 to 24 in each country have an unmet need for family planning, which contributes to the high percentage of women ages 15 to 19 who have begun childbearing (14.7% in Kenya, 19% in Nigeria, and 25% in Uganda) [14–16]. Across all three countries, the contraceptive method mix among modern contraceptive users ages 15 to 24 is unevenly distributed [17–19]. Short term methods (such as injectables, condoms, and pills) are commonly used among sexually active youth in all three countries, while unmarried sexually active women are less likely to use LARCs than their married counterparts [17–19].

HIPs that promote adolescent-friendly contraceptive services related to service delivery include non-judgmental service provision, audio and visual privacy, a wide range of available contraceptive methods, free or subsidized services, and community support [4]. This study focuses on Kenya, Nigeria, and Uganda because a systematic assessment of youth family planning policies and programming found that all three countries have a policy environment that supports at least two of these recommendations related to the service-delivery HIPs elements to improve youth-friendly contraceptive services [20]. Youth-friendly services (YFS) in family planning means providing youth with equitable, effective, acceptable, appropriate health services, since youth have needs that may not be met by standard health services [21, 22]. Despite favorable official policies, youth in these three countries continue to face barriers while accessing a full range of methods, including provider refusal and bias, limited contraceptive options, cost, and physical access constraints [23, 24]. These barriers also influence the decision-making process youth undertake when selecting a contraceptive method, the mechanisms of which warrant further study.

Understanding factors that influence youth to choose one method over another is important for effective implementation of policies and programs that aim to improve youth access to and use of contraceptives. Previous research has shown that a young person’s choice of a contraceptive method is a function of multiple processes, ranging from how youth collect necessary information, how they determine where and from whom to get the service, and how they cope with the uncertainty of whether they’ve made a comfortable and safe choice [25]. Picavet et al.’s model on contraceptive decisionmaking for women of reproductive age further organizes these processes with its inclusion of additional cognitive and social determinants [26]. The framework considers demographic factors, contraceptive and sexual background, individual values, decisional esteem, social influences, and environmental constraints. The model defines the contraceptive decisionmaking process as dynamic and dependent on one’s stage in life, experiences, situation, knowledge, and new information.

This qualitative study was conducted by PRB and International Youth Alliance for Family Planning (IYAFP) to assess gaps in implementation of YFS policies that prevent youth fro.m choosing from a full range of contraceptive options. Although the YFS policy landscape is promising in Kenya, Nigeria, and Uganda, the process of decisionmaking among youth as they navigate various barriers remains largely unknown. The study team applied Picavet et al.’s theoretical framework on contraceptive decisionmaking to group qualitative findings by relevant determinants of contraceptive method use among youth ages 15 to 24 in Kenya, Nigeria, and Uganda. The study aims to identify programmatic areas that decisionmakers and implementing partners can prioritize to improve implementation of existing policies that support youth access to and use of family planning.

## Methods

### Study design

We used two convergent qualitative approaches to capture young people’s and decisionmakers’ perspectives on youth access and use of contraceptives in Kenya, Nigeria, and Uganda: in-depth interviews (IDIs) with key stakeholders and focus group discussions (FGDs) with youth ages 15 to 24 [27]. The IDIs and FGDs used semi-structured question guides to balance consistency in the qualitative findings with flexibility to explore additional themes and topics. Before the start of data collection, both data collection tools were reviewed and validated by youth researchers, a group of youth family planning (FP) advocates, and select policymakers and subject matter experts in each country.

### Study setting and participants

Data collection took place in three study geographies in each country from October 2017 to December 2018. Study regions were selected using purposive sampling to include the national capital and two subnational entities. An additional table shows this in more detail [see Additional file 1]. To capture diverse experiences regarding youth access to and use of contraceptive services in differing sub-national contexts, we selected one sub-national entity with relatively strong sexual and reproductive health (SRH) outcomes for youth and one with relatively poor outcomes. SRH outcomes used to select subnational sites included teenage pregnancy rate, total fertility rate, median age at first birth, and median age at first sexual intercourse data taken from each country’s most recent Demographic and Health Survey. The team also took geographic accessibility and US Department of State travel restrictions into consideration. We selected the capital city as the third study geography in each country due to the variety of stakeholder types available and for the unique perspective of youth living in an area well-served by government FP programming.

IDI participants were recruited until data saturation was reached to represent a range of national and subnational stakeholders’ and decisionmakers’ experiences working in youth contraceptive services and related fields. Interviewees included national and state policymakers, program managers, service providers, community gatekeepers, and representatives of medical schools or other providers of clinician training, civil society organizations, and youth-serving organizations.

Youth participants were eligible for participation in the FGDs if they currently lived in the specific study setting, were between the ages of 15 and 24 (or 16 and 24 in the case of Nigeria, as explained below), and were sufficiently fluent in the spoken local language in which each FGD was conducted. Posters placed on community boards and in public areas frequented by youth, as well as a shareable electronic advertisement posted on Facebook, were used to recruit FGD participants ages 15 to 24. A separate poster advertisement recruiting youth between the ages of 15 to 17 was displayed in public areas where parents were likely to congregate and explicitly stated that parental consent was required for participation. Due to a lower age of consent for participation in research studies in Nigeria, the study only recruited youth ages 16 to 24 in that country.

### Data collection methods

We developed IDI and FGD interview guides informed by the seven HIPs elements of adolescent-friendly contraceptive services [see Additional files 2 and 3]. The guides also incorporated elements of the Health Policy Initiative’s Policy Implementation Assessment Tool, which considers policy understanding, dissemination, and utilization [28].

In each country, the data collection team consisted of two to three PRB staff with experience in qualitative data collection, a local consultant with YFS/SRH expertise, and three to four trained youth researchers.

In collaboration with youth researchers, PRB staff led interviews with key stakeholders in all three regions. The study team conducted the majority of IDIs in English, with a few exceptions when the interviewee felt more comfortable in their local language. Interviews averaged 45 min. Contemporaneous notes were taken by one member of the study team, and each in-depth interview was recorded with the participant’s permission.

Youth researchers from each country worked in pairs to conduct FGDs with youth participants in each of the six study geographies. Separate discussions were held for male and female participants and for minors (below age 18) and older youth (ages 18 to 24) (e.g.male 15–17, female 15–17, male 18–24, female 18–24). FDGs were conducted in the language(s) most comfortable for the participants, including Efik, Ejagham, English, Igbo, Luganda, Lusoga, Rukiga, and Swahili. On average, five participants joined each FGD; this group size was intended to facilitate meaningful and purposeful dialogue and gain insightful information from the participants [29]. Each discussion lasted approximately 90 min and was recorded. At the end of each FGD, the study team provided youth participants with the local currency equivalent of US$10 to compensate for their travel expenses to the focus group.

### Data management and analysis

Recordings of each IDI and FGD were transcribed and translated into English, if necessary, using notes to make the transcripts as complete as possible. Interview transcripts were imported into MAXQDA and reviewed to create a draft codebook based on the HIPs framework [30]. The codebook was refined and calibrated through test coding by multiple members of the study team, including youth researchers.

Each interview transcript was coded and analyzed along key themes. In each country, multiple members of the study team co-coded several transcripts together to ensure inter-coder reliability. Codes were continually assessed to determine their conceptual similarities and differences, and the codebook was adapted slightly as needed for each country. Finally, we used an adapted contraceptive decisionmaking framework to organize and analyze FGD and IDI data [26, 31].

### Ethics

Research ethics approval for the study was obtained from institutional review boards (IRBs) at Health Media Lab in Washington, D.C., AMREF in Kenya, National Health Research Ethics Committee in Nigeria, and the Makerere University School of Social Sciences Research Ethics Committee in Uganda. In most countries, once IRB approval was acquired, the study team obtained a letter of approval from the Ministry of Health that served to introduce the project to potential IDI participants and justify FGD proceedings. IDI participants completed signed informed consent forms prior to the initiation of each interview, with the exception of IDIs carried out in Kenya where IRB approval was not necessary for this portion of data collection. FGD participants ages 18 to 24 were informed of the study and were asked to indicate their consent by initials or a thumbprint. Parents or guardians of minors (ages 15 to 17) in Kenya and Uganda provided signed informed consent for their child to participate, and the minors participated only after assenting. In Nigeria, because the age of consent to participate in research studies is 16, parental consent was not needed for youth participants and all participants indicated their consent by initial or thumbprint.

## Results

We held 35 focus group discussions with 171 youth ages 15 to 24 across the three countries. We also facilitated 130 IDIs with national and subnational stakeholders, conducting between 42 and 45 IDIs in each country. The number of participants by country and data collection approach is shown in Table 1. We included both married and unmarried youth participants in FGDs where possible.

**Table 1:** FGDs and IDIs by number and participant type

	# of FGDs	# of FGD participants	# of IDIs	Total participants
Kenya	12	59	43	102
Female 15–17	3	15		
Female 18–24	3	14		
Male 15–17	3	15		
Male 18–24	3	15		
Nigeria	12	57	42	99
Female 16–17	3	14		
Female 18–24	3	15		
Male 16–17	3	16		
Male 18–24	3	12		
Uganda	11	55	45	100
Female 15–17	3	15		
Female 18–24	3	15		
Male 15–17	2	10		
Male 18–24	3	15		

**Fig. 1:**
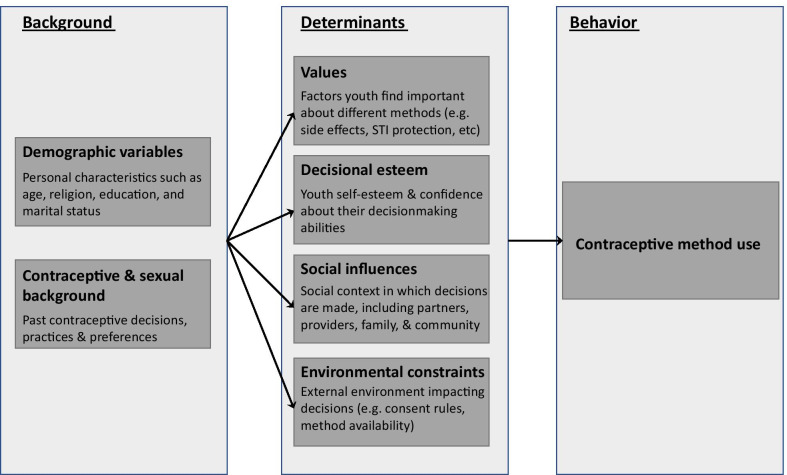
Adapted model of youth contraceptive decisionmaking

Figure 1 outlines the six components of Picavet et al.’s framework we used to organize results. These components most closely align with youth and stakeholder perspectives gleaned from our convergent qualitative data sources.

### Demographic variables: age and marital status jointly impact contraceptive choice

The Picavet et al. framework outlines how an individual’s demographic and personal profile comprises the basis of the contraceptive decisionmaking process. Youth in all three countries cited age, marital status, education level, religious beliefs, gender, and income level as determinants of their contraceptive choice. Age and marital status in particular emerged as major factors that impact young people in their contraceptive decisionmaking.

A majority of young people in all three countries reported that youth are denied certain contraceptive services if service providers perceive they are too young. Both youth and stakeholders noted that providers often ask young people to provide identification to prove their age or require them to obtain parental consent to access certain methods, even though official policies do not require it, as they are deemed too young to make that decision themselves. Younger youth ages 15–17 reported being discriminated on the basis of their age more than older youth ages 18–24.

*I have a 15-year-old friend at home. She had gotten pregnant at such an early age, aborted, got pregnant again, aborted. She was advised to get a long-term form of contraception. She went to a clinic but was told she was too young to make a decision whether to have a short- or long-term method. She had to be accompanied by someone who was allowed to make that decision—[someone] 18 or above, according to the Constitution.* (IDI, representative of a youth-serving organization; Nairobi, Kenya)

Although study participants in all three countries reported that service providers cite “legal age limits” when denying some contraceptive methods, particularly LARCs, without parental consent, national policies do not require it and some codify the rights of youth to obtain services without consent. Despite this, in Nigeria, providers’ insistence on consent makes it difficult for youth to obtain any contraceptives other than condoms. Most youth noted that parents are unlikely to give consent for their children to use contraceptives due to the cultural taboo around sexual activity at their age.

In all three countries, youth also reported that married youth can more easily access a wider range of contraception. Youth shared that key players in their social communities (such as parents, teachers, and community leaders) commonly believe that family planning is exclusively for use by married couples.

*Some persons have this mentality that family planning is for married people, so that is why it is difficult for them to support the younger person to go and do family planning.* (FGD, female, age 18 to 24; Cross River State, Nigeria)

They also explained that marriage often reduces the stigma young people face when accessing contraceptive information; young married people who would ordinarily face obstacles obtaining methods because of their age do not receive the same scrutiny from providers as their unmarried peers. Similarly, youth who go to health facilities with a partner report they are treated more favorably, presumably because they are perceived as married.

*You can go with your partner to get a method of contraception … and no service providers will ask you probing questions, as compared to going alone.* (FGD, female, age 18 to 24; Embu County, Kenya)

Findings indicate that, taken together, age and marital status can work in favor of youth access to contraceptives, if they align with pervasive cultural norms. For example, youth across the three countries unanimously reported that older married youth can more easily access a wider range of contraceptives, like injectables and implants.

Gender differences add another dimension to the impact of marital status on contraceptive access. Although marriage often insulates young women from the stigma that deters them from accessing contraception, married men may face increased stigma for seeking contraception.

*Now for a married woman who goes to get contraceptives, she will not be judged that much, even if she is buying a condom or even if she is buying a pill*. *But a married man who goes to buy condoms is perceived to be cheating on his wife.* (FGD, male, age 18 to 24; Abuja, Nigeria)

This was a reoccurring finding, demonstrating that youth consider the differential effects and interaction of marital status and gender in contraceptive decisionmaking.

Youth and stakeholders reported that young people’s income levels often influence their contraceptive options in all three countries. Youth participants reported that condoms are offered free, yet girls who cannot afford a method of choice tend to resort to traditional methods. These reflections were confirmed by stakeholders, who reported that if women do not have financial resources or empowerment, they may be unable to adopt or maintain contraceptive use. They also reported that when LARCs are only available for insertion or removal at expensive private facilities, such methods are often cost prohibitive to youth.

### Contraceptive and sexual background: availability still guides contraceptive practice and preference

The perceptions of youth and stakeholders about the range of contraceptive methods available to youth were often contradictory. Youth participants reported that method choice is often limited by the ease of access, the need for discreet use, and the options presented by service providers. Because of these external factors, in all three countries, youth reported that short-term methods are the most accessible contraceptive methods for them.

Youth noted that condoms are typically the most popular method among young people given their widespread availability and dual protection characteristics. Youth shared that condoms are usually placed where they can easily be accessed without interacting with a service provider who would otherwise subject them to questioning before providing their chosen method.

Additionally, youth may not be given an opportunity to fully consider a broader array of contraceptive options in a healthcare setting. Some youth were concerned that service providers working in health facilities over-emphasize condoms as a recommended contraceptive, deprioritizing or even declining to provide information on methods for young people.

*If service providers want to tell you which (method) to buy, they will just tell you about condoms first. Almost everybody knows about condoms.* (FGD, male, age 15 to 17; Cross River State, Nigeria)

Contrary to the reports of youth themselves, most IDI respondents asserted that youth are usually offered a full range of methods but choose short-term methods. They did acknowledge that, while service providers promote access to all methods for youth in individual consultations with patients, method choice is often limited due to either stock-outs or other factors, like lack of provider training to administer LARCs.

*We promote all methods, we don’t have methods that we specifically promote, and we train health workers to provide all methods. But the young people mostly take up the short-term methods than long-term methods.* (IDI, international NGO representative; Kabale, Uganda)

Apart from availability, other dynamics also played a role in youth contraceptive choice and preference. Younger youth ages 15 to 17 reported engaging in more unplanned sex, and therefore preferred short-term methods like condoms or emergency pills they found more convenient, compared to their married counterparts. Conversely, youth under parental guidance and those with non-supportive spouses chose more discreet methods like injectables:

*Girls prefer Injecta-plan [brand of injectable] because they believe they are safe for them and they save you the burden of keeping [a] bulk of pills, which a parent can bump into.* (Kabale FGD, female, 18 to 24; Kabale, Uganda)

### Values: youth prioritize protection and privacy and fear side effects

When information and access are not barriers, contraceptive choice is ultimately dependent on the factors that youth find personally important with regard to contraception.

In all three countries, youth acknowledged they use contraception to protect them from unwanted pregnancies, STIs, and HIV infections and to enable them to make informed decisions about their futures.

*Because if you don't get [contraceptives], she will get pregnant, and once she gets pregnant, you just know you have the responsibility; it's either to begin to prepare for the baby or prepare to abort a baby. The fear of the consequence will motivate you to either go get the condom or a pill.* (FGD, male, age 18 to 24 years; Abuja, Nigeria)

Youth participants also value contraception that is familiar. As condoms are most commonly used by young people, some respondents shared that they only transition to other methods after a condom has failed.

*For me personally, that [condom]'s the first thing you need—that is even basically the only thing you need. Thinking of any other form of contraceptive is like Step 2; that means Step 1 has failed you so you're thinking of a backup plan now.* (FGD, male, age 18 to 24; Abuja, Nigeria)

While young people in all three countries reported condoms as their preferred method, they also mentioned the importance of comfort and pleasure during sex when choosing a contraceptive method. They cited the discomfort or limited pleasure during intercourse associated with condom use as a reason for some resorting to withdrawal or safe days methods.

*Boys don’t like using condoms because they think that you can’t take sweets (experience sexual pleasure) in polythene bags, but the girls feel safe.* (FGD, female, age 18 to 24; Kabale, Uganda)

Some youth respondents shared that younger girls may prefer methods other than condoms for other reasons: either because they find them too much of a hassle to use or because they desire more discreet, undetectable methods like injectables.

Privacy is also an important factor in youth’s choice of contraceptive methods. Young people are afraid to be seen picking up or buying condoms and are even more embarrassed when seen accessing other methods. Young women also reported a desire for undetectable methods that help maintain their privacy at home. This influences what methods they choose and leads them to take protective measures when accessing methods.

*Mostly we go alone, but if it’s in an open place, we go with peers to surround the (condom) dispenser and we pick them. Or in the chemist, one person pays and the other collects to create some confusion.* (FGD, male, age 18 to 24; Narok County, Kenya)

Youth ability to protect privacy while accessing contraception determines their choice of service delivery points, as well as chosen methods. Both young people and stakeholders agreed that youth prefer going to private facilities because of more assured confidentiality compared to public facilities. Youth who prefer chemists for their ease and convenience are restricted to short-term contraception requiring continuous action and restricts access to information and counseling.

Youth frequently expressed fear of side effects from contraceptives, especially when using LARCs. They reported uncertainty around efficacy and future fertility, as well as fear of birth defects or excessive menstrual bleeding from using LARCs, and felt condoms are less harmful. They mentioned using information from friends who have used specific methods to help them choose a method or gauge its safety and efficacy.

*The experiences from my friends who have used these methods make me and other young people uncomfortable and reluctant to use family planning services, as they have reacted badly such that I cannot dare think of using these methods.* (FGD, female, age 18 to 24; Embu County, Kenya)

Stakeholders affirmed that young people would rather use short term methods instead of LARCs for fear of side effects, with an emphasis on future infertility.

*A key challenge to youth access is that … there is a fear of reversibility. ‘If I use an IUD, how will I get pregnant again?’ ‘If I use an implant, how will I get pregnant again?’ So people don’t want to use those long methods because they are scared that if they do, they will never get pregnant. This is a big source of concern for young people. This is a big concern here because once you get married, people expect you to get pregnant.* (IDI, international NGO representative; Abuja, Nigeria)

### Decisional esteem: access to  FP information and community norms present barriers

According to Picavet et al.’s model, youth’s autonomy to choose the right contraceptive method for them is determined in large part by their own personal esteem and by the information they seek out to help them in decisionmaking.

In focus groups, young people noted limited information about most methods of contraception except for condoms. Youth reported their main sources of contraceptive knowledge are the internet and peers, both of which often provide unreliable information. This is coupled with myths and misinformation about methods other than condoms, especially LARCs. Young people’s contraceptive decisions therefore draw on the incomplete information that is available to them.

*There is little information about contraceptive methods. For example, many people would be using female condoms if they knew how to use them. That is why we say that the most used contraceptive is the male condom, because people have a lot of information on how to use them.* (FGD, male, age 18 to 24; Mayuge District, Uganda)

The limited access to information about a full range of methods was also experienced during service provision:

*The counseling before one takes the method is critical. My impression is that where it hasn’t been done properly, young people are not told about the side effects. And after they experience side effects, they blame the method and develop bad opinions. Even though, in reality, they weren’t counseled and informed about what to expect, what side effects there may be, and how to manage the side effects. There is a missing link in the counseling for young people to adapt a method.* (IDI, NGO representative; Kampala, Uganda)

Even when youth are accurately informed about family planning, both youth and stakeholders agreed that social norms assign moral implications to sexual activity among unmarried youth, in turn impacting their access and choice. Most participants reported that the weight of this moral judgment tends to diminish youth decisional power.

*The message you hear everywhere is abstain from sex, no premarital sex. That is the option that seems to be given to young people. Why? Because it’s morally right.* (IDI, international NGO representative; Abuja, Nigeria)

Youth feel judged and demeaned when trying to access contraceptive information and products by service providers and others who violate their confidentiality and often aggressively question their decision to seek contraception. Youth reported being laughed at by their peers who discover they are using contraceptives. Embarrassment caused by the reactions of service providers and peers erodes young people’s confidence and self-esteem in their decisions to seek family planning services. Given such experiences, youth avoid accessing contraceptives in formal healthcare settings, even though they would have access to a wider range of options.

*The doctors, the nurses, the people youth meet at facilities discourage them. They will just start shouting, they just start judging you. And as medical personnel, it is not in your place to judge or ask those questions.* (FGD, female, age 15 to 17; Abuja, Nigeria)

Youth reported that they are not bold enough to walk into health centers to ask for information because many people believe that contraceptive knowledge is inappropriate for young people.

*I didn't even know there were injectables. And you walk into a pharmacy and ask for the female condom, the judgment alone is enough to kill you. It's just going to turn you off. Even before the idea of listening to the response you might just end up walking away.* (FGD, male, age 18 to 24; Abuja, Nigeria)

Decisional esteem was frequently developmentally specific to being young. Youth focus group participants directly commented on their need to hide their use of contraception for fear of being seen as immoral or as ungrateful kids, preventing them from seeking out the information they need to leverage more suitable contraceptive methods:

*We have those that will just assume this kid is spoiled, why should she start using [contraceptives] while she is so young?* (FGD, female, age 18 to 24; Nairobi, Kenya)

On the other hand, several youth reported knowing their rights and more confidently choosing any method they want without intimidation. Others acknowledged their aspirations to have high decisional esteem.

*The knowledge on the ease of getting the contraceptive is one burden, and the knowledge of using the contraceptive is another. But you find out that some people walk in with this euphoria—"I know what I want, and I know how to use it.”* (FGD, male, age 18 to 24; Abuja, Nigeria)

Factors impacting decisional esteem differed by gender. Youth reported that some young women must hide their contraceptive usage from both their partners and parents, while young men are not subject to this expectation. In such instances, young women are forced to make the contraceptive decision on their own and are limited to discreet methods.

### Social influences: stigma and fear impede accessibility to a full range of methods

Youth identified social stigma and engrained fear of community beliefs as the most common barriers to accessing any type of contraception. Young people in all three countries shared that they are growing up in communities and families that believe unmarried youth should not be having sex. This leaves youth feeling isolated, ashamed, worthless, and stigmatized.

*They (communities) think that you are spoilt, a prostitute. They don't even want you to be friends with their daughters because they fear you might spoil them also* (FGD, female, age 15 to 17; Mayuge, Uganda)

*There is a way they (parents) look at you when they see you with that condom pack and it just feels like you’ve messed everything up. So we are never comfortable when they see us with the condoms.* (FGD, male, age 15 to 17; Nairobi, Kenya)

In all three countries, youth reported stigma as the most important barrier to their comfort and positive experiences seeking contraceptive information or products.

At times, service providers allow their personal beliefs to override their ethics. In all three countries, while youth reported that some providers gave comprehensive information on a full range of methods, youth from each country also reported that some providers refrained from providing such information, and in some cases, actively discouraged youth from using LARCs:

*Youth use mostly condoms and emergency contraceptives. We are discouraged from using the long-term methods.* (FGD, female, age 18 to 24; Narok, Kenya)

Across all three countries, responses from youth and stakeholders indicate that persistent and pervasive myths and misconceptions ingrained in community norms and interactions played a significant role in how young people make decisions about contraceptives.

*Myths and misconceptions are a huge barrier (to contraception use)—that contraceptives will cause cancer, stroke, infertility, etc. Because of those myths and misconceptions, people even fear to demand or ask for a service. There are general myths and misconceptions for all methods, but it’s worse for LARCs.* (IDI, international NGO representative; Kampala, Uganda)

In societies that highly value children, the misconceptions around LARCs are usually related to fears of future infertility.

*The unmarried girls have more fears about the side effects [of contraceptives], they have misconceptions. Fear of infertility, fear of return to fertility. We are coming from a culture where there is high value on children and anything that will puncture that high value, people are ready to resist it.* (IDI, NGO representative; Abuja, Nigeria)

### Environmental constraints: challenges of contraceptive cost and availability

External factors, like cost, contraceptive stock-outs, and availability, also constrain youth contraceptive decisionmaking. Despite the fact that some countries offer subsidized or free contraceptives, commodities may be expensive or hard to obtain. Therefore, while cost and availability may be determining factors in contraceptive method choice and use, their impact is highly dependent on context.

Across all three countries, focus groups ranked financial barriers as secondary to the other barriers discussed above. Youth, in multiple settings, reported that they are willing to pay more for their preferred methods if possible and when warranted:

*I am not talking about affording it, I am talking about the best. … [T]here is no way you will want to go to the low [quality] one...[over] this one [that]…costs a little more.* (FGD, female, age 18 to 24; Nairobi, Kenya)

Other youth reported cost as secondary to their concerns of wanting to avoid poor quality or high stigma services, despite their lack of employment or income. For a majority of youth in our study, the “path of least resistance” in contraceptive decisionmaking means the path with less fear and discomfort if at all possible—even if it costs more.

Youth also discussed affordability and availability of contraceptive brands youth prefer to use. Just because a form of contraceptive is available doesn’t mean that the preferred brand or the healthcare setting in which it is available is affordable:

*We will always have the Durex [condom], but how many people can afford it?* (FGD, male, age 18 to 24; Abuja, Nigeria)

In focus group discussions, many youth highlighted that their preferred contraceptive methods are more expensive because they are largely available at private facilities rather than government or public facilities that provide free services.

Both youth focus group and IDI participants in our study reported that youth who were most impacted by contraceptive availability were more likely to be from rural areas. Additionally, youth in all three countries reported that certain commodities, such as implants and injectables, run out faster than others due to high demand, limited availability, or both. IUDs were generally reported to be as either limited in supply or unavailable across all three settings, with implants a close second. Condoms were more likely to face stock-outs due to high demand in all three countries.

In general, stakeholders in both Uganda and Nigeria commented that current contraceptive procurement processes, especially for LARCs, are not optimized to meet actual demand. Over- or under-projections lead to stock-out or excess supply.

*Certain commodities have been procured to capacity and they have been delivered to the end users, and the end users have not picked their choice. You find that some of [the commodities] have expired from the facilities and the end users are requesting for different commodities that have not been procured. There’s a gap in the supply chain.* (IDI, NGO representative; Kampala, Uganda)

In all three countries, stakeholders reported that stock-outs were usually limited to particular methods at different times and places due to demand. However, in Kenya and Nigeria, stakeholders reported a reduction of method choices further from the capitals, which was a unique dynamic in supply chain and commodity distribution contributing to stock-outs and limited access.

*The further you go out of Nairobi the harder it is to get all the choices; certain contraceptives are not accessible to certain areas, like LARCs.* (IDI, Government representative; Nairobi, Kenya)

While procurement and stock limitations affect all contraceptive users, they serve as an additional layer to the many barriers faced by youth.

## Discussion

Based on convergent qualitative data obtained from focus groups and individual stakeholder interviews, our findings illustrate despite favorable policies in Kenya, Nigeria and Uganda that support youth access to a wide range of contraceptive options, important factors prevent youth from making empowered decisions to choose their preferred method [12]. This may indicate that favorable policy environments alone will not be successful in the attainment of a broader method choice among young people. Contraceptive decisionmaking for youth is a dynamic process that is heavily influenced by external actors and bound by the local environment. These findings add to our understanding of the complexity of the pathways that youth must navigate to determine and obtain their preferred contraceptive method. Generally, the findings are in line with previous studies showing that adolescents’ and young people’s contraception choices are strongly affected by ease of access, the need for discretion, and service providers’ biases and attitudes towards young people and that short-term methods are used more commonly than LARCs [32–34]. In these three countries, youth report condoms as their preferred contraceptive method, which may be more a function of ease of access than empowered preference. In additional to availability, other factors such as unplanned sex and unsupportive parents and partners/spouses may also lead youth to choose condoms over other methods. Our findings suggest that, in their contraceptive decision-making, youth take the path of least resistance, which is a function of what is considered safe, available, known, and stigma-free, more than it is an empowered decision in which youth feel confident or supported.

For youth in the three countries and diverse geographies of this study, access to a wide range of family planning methods is limited by a set of common barriers: demographic factors, decisional esteem, social norms, provider bias, cost, and availability.

Among demographic factors, young people’s decisional processes and contraceptive choice are most strongly shaped by marital status [18]. While policies in these three countries do not restrict access to contraception based on marital status, the findings indicate that, irrespective of age, married youth are offered access to a wider range of contraceptives. Providers often adhere to cultural beliefs that unmarried youth are too immature to make the decision to have sex and that sex is a taboo act in which only married couples should engage. Focus group discussions demonstrated that non-judgmental service providers who respectfully provide comprehensive FP information and services to youth serve a protective role, but were mentioned more rarely by youth than judgmental providers.

While there are favorable policies in all three countries supporting access to a full range of contraceptives for youth, disharmony with other related policies and laws could explain some of the variance in service delivery and may be driving provider bias and youth reluctance to seek LARCs for fear of being identified as sexually active. In all three countries, the legal age for consensual sex is 18 years. However, the current policy environment in Kenya and Nigeria as of 2020 does not explicitly set an age for consent from a third party to access FP services, while Uganda currently affirms the right of youth of any age to access family planning without parental or spousal consent [35]. While disharmonization of related laws did not come out explicitly in our data, our findings show that consent was a major barrier to youth accessing more contraceptive options in all three countries.

Our findings suggest that it is incredibly challenging for youth to obtain the decisional esteem needed to make an empowered contraceptive decision as they navigate the interacting burdens of self-doubt and fear of failure, community or spousal retribution, and/or future infertility, in addition to fear of judgment and stigma from others, a related finding to other studies [18]. As a result, youth tend to seek contraception information and services from familiar sources that offer greater protection from scrutiny and prefer to skip the burdensome risk assessment required to deviate to new or more effective methods. Social reinforcement by informal peer networks likely explains the pervasiveness of misinformation and myths around certain contraceptive methods.

Consequently, condoms, emergency contraception, pills, and injectables remain the most commonly used contraceptive options among youth, since IUDs and implants require more intensive preparation and engagement with formal healthcare settings. This study suggests that equipping youth with skills that would enhance their self-esteem and assertiveness would improve their opportunities to access the full range of FP methods. Our findings suggest that peer networks serve as fundamental pathways for information-seeking for youth. However, research has demonstrated that peer education as a stand-alone intervention is largely ineffective in modifying behavior outcomes [36]. Moreover, youth-friendly FP programs limited to promoting use of condoms and other short-term methods that are readily available and accessible may seem unduly successful. Instead, improving the quality of information that youth receive from their preferred information channels about all contraceptive methods and redefining the role of peer educators as a referral to experts and services is likely to yield a more impactful youth-friendly service delivery system [37]. Our findings suggest that social influences play a pivotal role in shaping contraceptive decisionmaking for youth. Religious and cultural leaders predominantly promote abstinence for youth, and parents reinforce this social stigma. The findings suggest that providers’ desire to adhere to social norms can override their professionalism and compliance with YFS policies, causing them to offer biased information and incomplete contraceptive options to youth. This finding underscores the importance of ongoing provider training, especially around LARCs for young people, along with accountability mechanisms and incentives in ensuring high-quality YFS implementation.

## Recommendations

This study advises that favorable policy positions on youth-friendly family planning are alone insufficient to ensure youth achieve access to a full range of contraceptive methods. While policies may be strong on paper, there are other factors that influence how these policies are implemented, impacting the lived experience of young people who try to access contraceptives. Decisionmakers, administrators, and providers can improve implementation of YFS policies by addressing the complex, interrelated factors shaping youth decisionmaking. These efforts would ease the decisionmaking pathway for youth to make empowered choices, reduce barriers that often lead youth to choose the path of least resistance, and maximize the benefits of full method mix. The governments of Kenya, Nigeria, and Uganda need to support more robust investment in clarifying community values around contraception and addressing provider bias to improve youth access to a full range of methods. This includes proper sensitization of service providers to mitigate consent barriers based on heterogenous interpretation of discordant policies to position reproductive health/FP as a fundamental right to everyone, including youth. We also recommend extensive sensitization training for and information sharing with all community gatekeepers about the safety of all contraceptive methods for youth. These efforts could expand sources of accurate information and reduce the fear of fertility loss as a result of using some methods. Most importantly, to increase empowered contraceptive decisionmaking by young people, programmatic interventions should adopt gender transformative approaches and focus on expanding youth knowledge of the available supportive policies, contraceptive methods and side effects. Supporting young people’s confidence in their own decisionmaking will make their decisions more resilient in the face of complex barriers.

## Strengths and Limitations

Qualitative findings presented in this study were prone to social desirability bias and the sensitive nature of FP topics could have limited the sharing of information. The study also did not seek to make inferences from the FGDs based on a representative sample, but to capture broad and applicable themes related to youth experiences. However, the FGDs were conducted by local, trained youth researchers, which limit these bias concerns. The study team also aimed to capture more diverse voices in the FGDs by deploying a more inclusive recruitment strategy, including the use of poster advertisements and multiple visits by study research associates to recruit participants outside of major towns.

This study, as a collaboration between PRB, an international NGO, and International Youth Alliance for Family Planning, a youth-led organization, meaningfully engaged youth at every stage, from instrument development to data validation. Additionally, we were able to capture perspectives disaggregated by age, location, and gender, allowing us to make comparisons within and across groups. Lastly, convergent qualitative methods between FGDs and IDIs and triangulation of these findings provided richer context for young people’s perspectives and contraceptive decisionmaking experiences.

## Conclusions

Efforts to strengthen the policy environment for youth-friendly contraceptive services have been successful in Kenya, Nigeria, and Uganda, but demographic factors, contraceptive and sexual background, individual values, decisional esteem, social influences, and environmental constraints still prevent youth from choosing from a full range of contraceptive methods. Young people’s contraceptive decisionmaking is heavily influenced by a desire to avoid a multilayered set of obstacles associated with youth contraceptive use. These desires result in young people taking the path of least resistance, which most often leads youth to choose condoms. Those responsible for YFS policy implementation can expand youth knowledge of contraceptive methods and side effects to support their confidence in their own decisionmaking; address community and provider beliefs that youth use of contraception should be conscribed based on age and marital status; cater to distinct differences between what young people find important about contraception use and what older users do; and ensure expanded accessibility of facility-based services for youth.

## Supplementary Information

**Additional file 1.** Study Regions in Each Country. Detailed information on study regions in each country and reason for selection.

**Additional file 2.** IDI questionnaire. A version of the questionnaire that was used to interview key stakeholders in all three countries. For each country, the wording and order of certain questions were altered based on context.

**Additional file 3.** FGD questionnaire. A version of the questionnaire that was used to interview youth participants in all three countries. For each country, the wording and order of certain questions were altered based on context.

## Data Availability

The data that support the findings of this study are available from authors at Population Reference Bureau, upon reasonable request.

## Abbreviations

FP: Family planning
FGD: Focus group discussion
HIPs: High-impact practices
IDI: In-depth interview
IUD: Intrauterine device
IYAFP: International Youth Alliance for Family Planning
LARC: Long active reversible contraception
NGO: Non-governmental organization
PRB: Population Reference Bureau
SRH: Sexual and reproductive health
SSA: Sub-Saharan Africa
YFS: Youth-friendly services

## Footnotes

1 This study uses the terms “youth” and “young people” interchangeably to mean those 15 to 24 years old.
