# Supplementary material for: A cross-country qualitative study on contraceptive method mix: contraceptive decisionmaking among youth
Source: Reprod Health. 2021 May 25;18:105. doi: 10.1186/s12978-021-01160-5 (PMC8145785; doi:10.1186/s12978-021-01160-5)
Supplement: Supplementary file 1 — Additional file 1. Study Regions in Each Country. Detailed information on study regions in each country and reason for selection. [file 12978_2021_1160_MOESM1_ESM.pdf]

Study Regions in Each Country

	Capital	Subnational entity with poor youth SRH outcomes	Subnational entity with strong youth SRH outcomes
Kenya	Nairobi	Narok County	Embu County
Nigeria	Abuja	Cross River State	Anambra State
Uganda	Kampala	Mayuge District	Kabale District
Note: SRH outcomes used to select subnational entities included teenage pregnancy rate, total fertility rate, median age at first birth, and median age at first sexual intercourse. The team also took geographic accessibility and U.S. Department of State travel restrictions into consideration. The study team assessed SRH outcomes based on the breakdown of data in each country’s most recent Demographic and Health Survey (DHS). While Uganda is divided administratively into 112 districts, the DHS groups them into 15 regions. The study team then selected a single district within each region to ensure data collection was contained within an official governing zone.			
