# Supplementary material for: A cross-country qualitative study on contraceptive method mix: contraceptive decisionmaking among youth
Source: Reprod Health. 2021 May 25;18:105. doi: 10.1186/s12978-021-01160-5 (PMC8145785; doi:10.1186/s12978-021-01160-5)
Supplement: Supplementary file 2 — Additional file 2. IDI questionnaire. A version of the questionnaire that was used to interview key stakeholders in all three countries. For each country, the wording and order of certain questions were altered based on context. [file 12978_2021_1160_MOESM2_ESM.pdf]

Key Informant (In Depth Interview) Assessment Tool

Note: Only select questions will be asked to each interviewee based on their experience, expertise, and time available for the interview. The numbered questions are priority questions, and each bullet point is a follow-up/probe that will only be asked if appropriate.

General Questions

1. Can you briefly share activities your department (or organization) implement related to contraceptive services for youth?
    - What are some highlights from these activities from the last year?
    - How is your department (or team) measuring the progress, effectiveness, or impacts of these activities?
    - Are there any challenges that you are seeing in implementing these activities?
    - Do you anticipate any future challenges in implementing these activities?
    - How do these (activities) fit in with national policies? Which policies?

2. In your opinion, do existing policies and guidelines commit to providing youth-friendly contraceptive services? Which of these policies are you familiar with?

    - [List country specific policies]

    [Focus questions 7 & 8 to top two policies respondent is familiar with]

3. Where and how are youth receiving information about family planning in [country]?
    - [Probe for family life and comprehensive sexuality education] What is the current status of the national/regional comprehensive sexuality education curriculum?
    - Has this program been effective at reaching youth with information?
    - What is the focus of SRH education in schools?
    - How are those youth that are out of school receiving information about contraception?
    - [Probe for mass media IEC campaigns] Do mass media campaigns exist that reach youth with information about contraceptive services?
    - [Probe for youth forums] Do youth forums exist that reach youth with information on contraception?
    - Have these campaigns been effective at reaching youth with information?
    - To what extent do youth face barriers in getting comprehensive information on contraception and family planning? What are the solutions to overcoming these barriers?

4. Generally, when youth seek contraceptive services in [country], where do they seek them?
    - What type of facility do they tend to go to? Probe: public vs private; clinic or chemist; schools; NGOs? Probe: Why do they prefer one over the other?
    - What contraceptive services exist at the community level for youth? (probe: such as through community health units/dispensaries or community health workers, mobile outreach)

"Elements of Youth-Friendly Contraceptive Services Provision" Questions

Build an Enabling Legal, Political, Social and Financial Environment

5. Is there political will to provide contraceptive services to youth by both national and subnational leadership?
    - Does political will differ among leaders at the national level as compared to the state or local levels?
    - To what extent has political will resulted in changes in the implementation of contraceptive services for youth?
    - *If political will is <u>not</u> strong, then ask:* Why is there a lack of political will? And what needs to be done to bring about/strengthen political will?
    - *If political will <u>is</u> strong, then ask:* What specific actions or strategies have been employed to provide contraceptive services to youth?

6. Have national policies regarding family planning services and providing youth friendly health services been disseminated? [Focus on top two policies from question 1]

    - How have they been disseminated?
    - Is there a plan for dissemination of these policies? Which levels of the health system are targeted? [Probe: Have these policies been disseminated to the facility and provider level?]
    - Have you seen any challenges in disseminating these policies? If yes: What are your recommendations for overcoming these challenges?

7. Are there strategies and protocols in place at the national level that translate policy provisions on contraceptive services for youth into actionable steps for implementation? If no, why not? If yes, what are the strategies and protocols in place? [Focus on top two policies from question 1]
    - Are there barriers to implementation? [Probe: Is there clear understanding of [and agreement on] how to implement these policy provisions?]
    - Are implementation plans being created?
    - Are there plans to evaluate the implementation of these policy provisions?

8. Is there a dedicated budget allocated for youth friendly contraceptive services at national/state levels?
    - If NO, what are barriers to creating a dedicated budget line?
    - If YES, has funding for contraceptive services for youth been successfully released/designated in each of the last two years?
        - If not: Why hasn't the budget allocation been released? How can budget allocation be improved?

9. Are funds for contraceptive service provision for youth tracked to ensure they are spent as allocated?
    - If YES, what methods are used to monitor these funds? How could tracking mechanisms be improved upon?
    - If NO, what are the reasons for failing to track these funds? What are potential solutions to overcoming these reasons?

10. Have youth participated in the design, implementation, and evaluation of contraceptive service delivery activities targeting youth?
    - If yes: how has this happened?
    - If no: Why have youth not been involved/ what are barriers to securing their involvement?
    - In what ways will they be engaged in the future?

11. Are youth ages 15-24 able to access contraceptives regardless of [age, marital status]?
    - If yes: What measures are being taken to ensure this access?
    - If no: What are the barriers/challenges to doing so? What are solutions to overcoming these challenges?
    - Are they able to access contraceptives without their spouse or parents' consent or presence? Probe: To what extent are youth supported by parents or spouses to use family planning?

12. Do guidelines/directives exist that require providers to deliver family planning services to youth without discrimination or bias?
    - In practice, do providers deliver family planning services to youth without discrimination or bias?
        - If no: What are the barriers/challenges to doing so? What are solutions to overcoming these challenges?
        - If yes: Are there specific examples you can think of providers offering FP services without discrimination or bias?

Provider Training and Support and Facility Processes:

13. Are there organized efforts underway to train providers to offer youth-friendly contraceptive services? If so, what does that organized effort entail?
    - How regularly are the trainings occurring? Are they repeated periodically? Are they occurring in all [zones/states/localities?] Has the next training been organized? Do you know of any trainings that have happened in the last six months?
    - Besides providers, are health managers being trained? Are administrative staff at facilities being trained?
    - What are the current challenges in providing training in youth-friendly services?
    - What solutions do you recommend to address these challenges?

14. What does the training include/entail for service providers that are being trained to offer youth contraceptive services?
    - Which training manual or program, if any, do you use?

- What curriculums are available to use for provider trainings on youth-friendly contraceptive service provision?
- What are specific skills for working with youth that are emphasized in the training?
- How are providers trained to communicate with youth who are considering uptake of contraceptive services?
- How are providers trained to withhold judgment and bias?
- Are there gaps in the content of the training? If so, what are these gaps? How would you recommend these gaps be addressed?
- To what extent do providers who undergo training apply this knowledge to interactions with youth seeking contraceptive information and services?

15. Can you speak about the length of time providers trained in youth-friendly contraceptive services stay in their current positions?
    - In your experience, has retention of these staff been an issue when attempting to train providers in youth-friendly services?
    - What are the challenges in retention? What solutions do you recommend to address these challenges?
    - In what ways are providers that are trained in youth-friendly services motivated to stay in their current position? What are the challenges to motivating staff? What solutions do you recommend to address these challenges?

16. Is there a system in place to supervise and monitor providers that have been trained?
    - What are those monitoring and supervision systems?
    - How do you ensure providers apply the knowledge learned from the trainings when providing contraceptive services to youth?
    - What are the monitoring and supervision challenges? What are the solutions you recommend to address these challenges?

17. Can you think of any programs or facilities that are doing an exceptional job at providing contraceptive services tailored to youth?
    - Are they located in a rural or urban setting?
    - What are they doing (differently) that makes them an exception program or facility compared to other programs or facilities? [we hope to hear about: special opening hours, privacy, confidentiality, method mix, trained providers]
    - Do these serve as an example to other facilities? [we hope to hear about joint learning]

18. Suppose that a health facility wanted to be more youth-friendly in their service provision. How would they go about that?
    - Alternative: Suppose a health facility or provider wanted to receive youth-friendly training. What is the process to receive YF training from the government?
    - Are there challenges in these processes? If so, what? How can these processes be improved?
    - How does the process differ for offering contraceptive services to youth outside of the health facility in other venues/places? What, if any, are there challenges in starting up

these services outside of health facility settings? What are the solutions to these challenges?

Enforce Patient Confidentiality and Privacy:

19. What measures are in place to ensure youth have full privacy and confidentiality when accessing contraceptive services?
    - Service Provider: How do you ensure privacy and confidentiality of the services you provide? Were you ever trained to do so? [Probe: safe storage of client records and such as nondisclosure of adolescents' health information]
    - Provider: To what extent is your facility equipped to ensure privacy? If so, how? [Probe: such as private screening and counseling rooms and partitioned waiting areas]
    - Youth-led org: In your experience, are facilities equipped to ensure privacy?

Offer a Wide Range of Contraception:

20. What contraceptive options are offered to youth [nationally/in the state]?
    - Are providers hesitant to provide certain contraceptive methods to youth?
        - If yes, which methods? Why is this so?
        - What are provider myths and misconceptions surrounding certain types of contraceptives?
        - [If long-acting methods are not mentioned, ask a follow-up question about LARC methods]
    - Can youth obtain both short-term and long-acting methods (e.g. IUCD or implant)? If no, why not?
    - Are there guidelines in place that instruct providers to offer both short-term and long-acting reversible contraception to youth?
    - Are steps being taken to increase providers' skill and comfort levels to provide long-acting reversible contraceptive methods to youth?
        - If so, what steps?
    - How can youth increase access to a wide range of contraception be improved?

21. In general, are youth counseled about contraceptive options at public facilities, private facilities, and chemists?
    - Does this counseling include a range of options with full informed consent? What about accurate information?
    - In general, does the quality of counseling differ among these facilities?
    - What are barriers to youth receiving high quality counseling on contraceptive options? What are solutions to overcoming these barriers?

22. Are you aware of any supply chain problems or commodity quality issues that affect SRH commodities used by youth in particular? [probe: stock-outs, expired drugs]
    - If yes, are certain commodities more affected than others?

        If yes, how are those problems being addressed?

- Can you tell us about mechanisms in place to prevent stock-outs of contraceptive commodities most used by youth? [e.g. commodity forecasting]
- *If respondent discusses commodity issues, then ask*: Do contraceptive commodity availability issues impact youth more than the general population?

Provide No-cost or Subsidized Services

23. Are contraceptive services provided free of cost or at a reduced cost for young people?
    - If so, what programs are in place (/by what organizations) to offer youth reduced cost or free contraceptives and contraceptive services? [Such as social marketing, vouchers, social health insurance.]
    - Do youth think that contraceptive services are "affordable" to them? [this is a less leading way of asking: Are youth citing cost as a barrier to accessing contraceptive services?]
    - If no, what challenges have there been in providing services to youth at an affordable cost? What are the solutions you recommend to address these challenges?

    - cost? What are the solutions you recommend to address these challenges?

Build Support in Communities for Youth SRH

24. Are there challenges in changing community opinions about youth contraceptive services? If yes, what are these challenges and what are the recommendations to address them?

25. Are there community-level programs in place to educate parents, community members, and community leaders about contraceptive services? If so, what are they?
    - Who is conducting these programs/activities? [Probe: government agencies, NGOs, etc.]
    - Since the implementation of these programs, to what extent have community members' opinions and behaviors surrounding the provision of contraceptive services to youth changed?
    - What are the challenges in implementing these programs?
    - If there are no programs in place, do you recommend that these or other efforts be put in place to build community support for youth contraceptive services?

26. To what extent is mass media reaching communities with information about contraceptive services for youth?
    - Which forms of media and what as the geographical scope?
    - What is the content of these messages?
    - Since the implementation of these campaigns, to what extent have community members' opinions and behaviors surrounding the provision of contraceptive services to youth changed because of the contribution of the media?
    - If these messages have not changed community opinions on youth contraceptive services, why not?

27. To what extent are there youth-led programs in place to educate and build support for youth contraceptive services among peers?

28. Have males been engaged around the issue of youth access to contraception? If yes, how?
    - Who does this include? [Probe: husbands, partners, fathers, male opinion leaders, husbands' family?]
    - What have been the outcomes from engaging these men?
    - Are young males targeted in youth sexual and reproductive health education and outreach?

29. In your opinion, which has been the most effective method of building support in communities for youth SRH and why?

Address Gender and Social Norms

30. Do gender and social norms affect youth access to contraception?
    - [explain "gender norms" and "social norms" as needed for the interviewee]
    - What are some of these social norms affect access to contraception among youth in [country]? Why do they affect access to contraception among youth?

31. In your experience, what is considered appropriate family planning messaging to adolescents and youth in [country]? [Probe: sexual abstinence]
    - Does this viewpoint pose a challenge for youth accessing contraception? What are the recommendations to address these challenges?

32. Do young men and women have different experiences when accessing contraception? If so, how? What are some examples of this?
    - Do certain groups, such as unmarried young women, have different experiences when attempting to access contraception? What about young married women who want to access contraception? What about young mothers?

33. To what extent are these gender and social norms being addressed in order to make it easier for youth to access contraception?

34. What are the challenges to addressing these gender and social norms? What do you recommend to address these challenges?

Conclusion

35. If you were to advise a group of youth advocates about where to focus their advocacy work in order to improve youth access to and use of contraceptive information, supplies, and services, what advice would you give?
