# Supplementary material for: A cross-country qualitative study on contraceptive method mix: contraceptive decisionmaking among youth
Source: Reprod Health. 2021 May 25;18:105. doi: 10.1186/s12978-021-01160-5 (PMC8145785; doi:10.1186/s12978-021-01160-5)
Supplement: Supplementary file 3 — Additional file 3. FGD questionnaire. A version of the questionnaire that was used to interview youth participants in all three countries. For each country, the wording and order of certain questions were altered based on context. [file 12978_2021_1160_MOESM3_ESM.pdf]

Focus Group Discussion Guide

Introduction

Thank you again for participating in this study. Let me begin by introducing myself [introduction]. I'd also like [name of other research associate and study team member(s)] to introduce themselves [introduction]. Together, we are collecting data to understand and assess the implementation of policies and their full impact on young people's access to and uptake of family planning information and services. We are having discussions like this with several groups around the country.

You were invited because as youth, you are directly affected by government policies surrounding access to FP. Please feel free to speak openly and use any language or words that you are comfortable using. There are no right or wrong answers. We encourage you to have varying opinions, and having a different opinion will not make you feel bad, will not make you look bad, or affect you in any way. You can speak in [relevent language(s)]. Again, you can choose to stop participating in this discussion at any time and you can also choose to not respond to any questions you don't want to answer. We are interested in hearing about youth experiences. You can choose whether or not you would like to share your personal experiences at any time. You are not obligated to share any personal stories, beliefs, or practices that you are not comfortable sharing.

Each of you will be provided with the monetary reimbursement for your time and travel expenses after the discussion ends. If you choose to leave the discussion before it ends for any reason, please try to leave without disrupting others, and you may collect your reimbursement from [name of other research associate.]

You've probably noticed the microphone. We're audio recording the session because we don't want to miss any of your comments. People often say very helpful things in these discussions and we can't write fast enough to get them all down. We will be on a first-name basis today (can be any name you choose) and we won't use any actual names in our reports or articles. You may be assured of complete confidentiality. We ask that you please not share any of the comments anyone in the room shares with others who were not present after the discussion. The results from this study will be shared online and with decision makers, CSOs, and governments to help improve implementation of policies.

Let's begin. Remember, it's important to speak one at a time and refrain from interrupting others. The facilitators will also make sure that everyone gets a chance to contribute to the conversation.

Any questions? [Pause] If there are no questions, we will turn on the recorder right now.

Icebreakers

1. If you could be any animal, which animal would you choose to be?

2. What activities do you enjoy doing at home? What activities do you enjoy doing outside of your home?

3. Suggested Icebreaker – Toilet Paper "Favorite Things" Introduction. Each participant in the FGD (including participants) takes out 1-5 toilet paper sheets from one roll of toilet paper being passed around. Each participant then shares their favorite thing (favorite music artist, color, song, etc.) themselves according to the number of toilet paper sheets they have taken out. The more the number of sheets, the more favorite things they share.

General sexual, contraceptive, and reproductive health service knowledge

1. What are the different contraceptive methods for young people that you know of?
   Probe: Specific methods (IUD, implant, pill, injection, ring, condom, calendar, birth control pill, after-sex pill , etc.)
   Probe: What do you know about these methods?

2. How and where do you learn about sexual and reproductive health information?
   Probes: School (sexuality education program), friends, parents, mentors, television/radio, etc.

3. What health facilities in your area are known for providing reproductive health services to youth?
   Probes: Local clinics, major names (ex. MSI), public vs private clinics

Experience with FP services

1. Tell me about your experiences using the healthcare system in [country] for reproductive health. (healthcare system: *hospitals, clinics, mobile clinics, pharmacies, doctors, nurses, payment methods, public vs private clinics, etc)*
   Probe: Are you able to get the service you need at the time you need it?
   Probe: What type of healthcare provider do youth usually go to?
   Probe: What is the process for paying for services?
   Probe: What level of facility do you usually go to?
   Probe: Who usually accompanies youth when they seek services?
   Probe: How do services differ at different levels? How is quality different at different levels? ? Is there a difference in offerings or quality in public versus private?

2. How do youth seek family planning services in your community?
   Probe: If somebody needed contraception, who do they ask and where do they go?

Probe: How do they get there?
Probe: How do they afford it?
Probe: What is the most ideal situation?

3. How would you describe what it is like for young people when they use family planning services?
   Probe: How comfortable do youth feel seeking family planning services?
   Probe: What emotions do youth feel as they seek family planning services?
   Probe for: satisfaction level, anxiety, embarrassment, happiness, empowered
   Probe: Do you share your experiences with your friends? Romantic partners?

4. What are some things that youth like/dislike about seeking family planning services?
   Probe: what are some of the things that prevent youth from seeking FP for services? What are some of the things that make youth more likely to want to go for FP services?

5. What do you think the most important family planning services are for young people in this community?
   Probes: contraceptive counseling, STI testing clinics, informative workshops, etc

6. What family planning methods are usually offered to youth?
   Probe: Are you/youth satisfied with the methods available?
   Probe: Are there any problems with stock outs or quality of the contraceptives?

7. Is there a difference in quality and experience in FP services between men and women? What about between married and un-married individuals? What about for youth of different ages?
   Probe: rejection of services, comprehensiveness in contraceptive counseling, coercion with method use, any instances of parental or spousal consent being required
   Probe: In your experience, are male and female service providers available to attend to you when you want?

8. How would you describe the knowledge of providers to give FP services?
   Probe: When you go for services, do providers tell you about your FP options?
   Probe: When you ask FP questions, are you satisfied with the answers given?
   Probe: Are you satisfied with the providers skillset to administer the FP method?

9. How friendly are the providers and staff where youth get FP services?
   Probe: Are you satisfied with how providers are communicating? Are you satisfied with your treatment? (Assess for provider bias/judgment)
   Probe: when youth go for FP services, in what ways do providers ensure confidentiality?

10. Do you feel that the facilities where you seek FP services are suited to your needs?
    Probe: Are they affordable?
    Probe: Do they have convenient opening hours?

Probe: Are there things to do in the waiting room?
Probe: Are facilities equipped to ensure privacy to youth clients?

11. Are there certain members of the community that are supportive of youth having access to contraceptive services? Who are they? Why?
    Probe: How are they supportive?
    Probe: Are there certain members of the community who are unsupportive of youth having access to contraceptive services? Who are they? Why?
    Probe:How are they unsupportive?
    Probe: men, fathers, husbands, community leaders, mothers-in-law, religious leaders, peers, community health workers

Knowledge of country's policies

1. Have you noticed any changes to the overall healthcare system in the last year? Three years? Five years?
   Probes: new reproductive health policies passed that look to serve adolescents regarding accessibility, availability, affordability, quality of services

2. What do you already know about [list relevent policies]?
   Probes: What does it include? Does anything include young people? What sections talk about FP?
   If they know some information:
   Probes: What are the channels for young people to learn about the [policy x]? Where did you learn about it?

3. What do you know about FP2020 and commitments that your country has made to family planning?'

4. Are there FP services that are illegal or not permissible for youth in your country?

5. In your opinion, how have government and local stakeholders engaged and involved youth in their decision-making processes to improve family planning services?

Opportunities for change

Thank you so much – we are almost done! I'd like to ask you now about FP service delivery and improvements that can be made in your community.

1. What do you think is the most important change that should be made so that FP services are easier for young people to access and use?
   Probes: Finances, number/accessibility of service delivery points, types of service delivery points, method mix, provider behavior, confidentiality, comprehensive sexuality education, parent/community engagement

2. Are there any health services or contraceptives you think youth need but are not available? (either they do not exist in the country or they are not available to youth)

3. What would you improve to ensure youth-friendly family planning service provision in your community?
   Probes: Adolescent-friendly contraceptive services, Enforced confidentiality and audio/visual privacy, wide range of contraception, no-cost/subsidized services, feeling safe in a positive legal and political environment

Wrap-up

Thank you very much for taking the time to speak with me today. Those are all the questions I have.

Here's a short summary of what we've discussed today [summary].

1. Is there anything that you would like to add?

2. Is there anything that I should have asked but didn't?

3. Do you have any questions for me?
